# ADAMTS9, a member of the ADAMTS family, in Xenopus development

**DOI:** 10.1016/j.gep.2018.06.001

**Published:** 2018-09

**Authors:** Ines Desanlis, Hannah L. Felstead, Dylan R. Edwards, Grant N. Wheeler

**Affiliations:** a School of Biological Sciences, University of East Anglia, Norwich Research Park, Norwich, NR4 7TJ, UK; b Norwich Medical School, University of East Anglia, Norwich Research Park, Norwich, NR4 7TJ, UK

**Keywords:** ADAMTS9, *Xenopus laevis*, Xenopus tropicalis, ECM, Versican

## Abstract

Extracellular matrix (ECM) remodeling by metalloproteinases is crucial during development. The ADAMTS (A Disintegrin and Metalloproteinase with Thrombospondin type I motifs) enzymes are secreted, multi-domain matrix-associated zinc metalloendopeptidases that have diverse roles in tissue morphogenesis and patho-physiological remodeling. The human family includes 19 members. In this study we identified the 19 members of the ADAMTS family in *Xenopus laevis* and *Xenopus tropicalis*. Gene identification and a phylogenetic study revealed strong conservation of the ADAMTS family and contributed to a better annotation of the *Xenopus* genomes. Expression of the entire ADAMTS family was studied from early stages to tadpole stages of *Xenopus*, and detailed analysis of ADAMTS9 revealed expression in many structures during organogenesis such as neural crest (NC) derivative tissues, the pronephros and the pancreas. Versican, a matrix component substrate of ADAMTS9 shows a similar expression pattern suggesting a role of ADAMTS9 in the remodeling of the ECM in these structures by degradation of versican.

## 1 Introduction

**Fig. 1:**
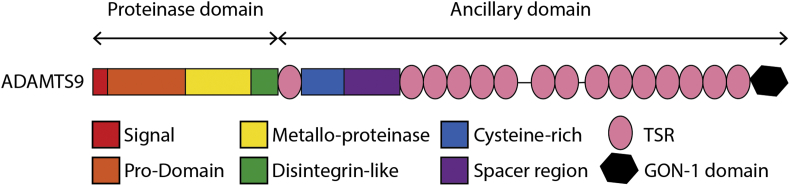
Molecular structure of ADAMTS9. The basic ancillary domain has a central TSR, a cysteine-rich domain and a spacer region. The carboxy (C)-terminal region of the ancillary domain is the most variable between the ADAMTSs containing one to fourteen additional Thrombospondin type 1 Sequence Repeat (TSR) motifs and specific domains defining different sub-groups. Names of the different domains are indicated below the diagrams.

The A Disintegrin and Metalloproteinase with Thrombospondin type-1 motifs (ADAMTS) family are extracellular, secreted enzymes that have diverse functions in development and disease. Nineteen members are present in mammalian genomes. The evolutionary history of the 19 mammalian ADAMTS genes has been marked by duplication and retrotransposition events giving rise to different subfamilies, in association with the organism's complexity, creating neofunctionalization. All ADAMTS proteins have the same basic domain organisation defined by a proteinase domain and an ancillary domain (Fig. 1) (Kelwick et al., 2015). The N-terminal region comprises an amino (N)-terminal signal peptide followed by the proteinase domain which includes a pro-domain and a catalytic domain containing the metalloproteinase domain and the disintegrin-like domain. The basic ancillary domain has a central Thrombospondin type 1 Sequence Repeat (TSR), a cysteine-rich domain and a spacer region. The differences in domain organisation and function define eight sub-groups of ADAMTSs: the hyalectanases/aggrecanases (ADAMTS1, 4, 5, 8, 9, 15 and 20), the procollagen N-propeptidases (ADAMTS2, 3 and 14), the von-Willebrand Factor (vWF) cleaving protease (ADAMTS13), the Cartilage Oligomeric Matrix Protein (COMP) proteinases (ADAMTS7 and 12) and the “orphan” (ADAMTS6, 10, 16, 18,17 and 19). The C-terminal region of the ancillary domain is the most variable between ADAMTSs, except in ADAMTS4 the spacer domain is followed by additional TSR domains. The ADAMTS9, 20 pair contains 14 additional TSRs and a gonadal (GON) domain in C-terminal. ADAMTS13 has two CUB (complement C1r/C1s, Uegf (epidermal growth factor-related sea urchin protein), BMP-1 (bone morphogenetic protein-1)) domains in addition to 7 additional TSRs. Several ADAMTSs (ADAMTS2, 3, 6, 7, 10, 12, 14, 16, 17, 18 and 19) contain a PLAC (protease and lacunin) domain. In the pro-collagen N-propeptidases (ADAMTS2, 3 and 14) sub-group the PLAC domain is embedded within a region unique to these enzymes, while in all others the PLAC modules mark the C-terminus of the proteins. In ADAMTS7 and 12, a mucin/proteoglycan domain is interposed between the 7 additional TSRs, precisely between TSR4 and TSR5 (Fig. 1) (Kelwick et al., 2015).

The largest subgroup of the ADAMTS family is the aggrecanases/hyalectanases comprising ADAMTS1, 4, 5, 8, 9, 15 and 20. These enzymes are so-called because they are able to cleave members of the hyalectan (also known as lectican) family of hyaluronan-binding chondroitin sulfate proteoglycan (CSPG) extracellular proteins, which include aggrecan, versican, brevican and neurocan (Stanton et al., 2011). Hyalectanases play diverse roles during development. *Adamts9* gene-trap mutant (Gt) mice, where ADAMTS9 is restricted to the cell surface due to the insertion of the human Bcl2α (a mitochondrial membrane protein) at the C-terminus, developed normally up to gastrulation but then showed increasing developmental defects suggesting different functions for ADAMTS9 dependent on whether it is localised to the cell surface or secreted (Nandadasa et al., 2015). It has been shown that ADAMTS9, 20 and 5 have non-redundant and cooperative roles in interdigital web regression by degrading versican (Dubail et al., 2014) (McCulloch et al., 2009b). ADAMTS5 and 9 are essential for heart development by cleaving versican (Dupuis et al., 2011) (Kern et al., 2010). ADAMTS20 mutant mice (belted, *bt*) have a distinctive coat with a white belt in the lumbar region suggesting the role of ADAMTS20 in the development of skin pigmentation (Rao et al., 2003). This phenotype is stronger when they are also heterozygous for an Adamts9 null allele suggesting cooperation between ADAMTS9 and 20 for melanoblast survival (Silver et al., 2008) (Rao et al., 2003). The double mutated mice (*Adamts9+/−*; *bt/bt*) have a cleft palate associated with decreased versican cleavage and reduced cell proliferation in the palatal shelves suggesting that versican proteolysis is necessary for cell proliferation in the fusing palate (Enomoto et al., 2010).

The development and the morphology of most organs are conserved between Xenopus and mammals, which makes it a good model to study developmental processes such as extracellular matrix (ECM) remodelling by the ADAMTS family. Very little is known about the ADAMTS family in Xenopus. The 19 ADAMTS genes were identified in *Xenopus tropicalis* and ADAMTS1 was shown to play a role in *Xenopus laevis* development at blastula to gastrula stage as a negative regulator of FGF by its C-terminal region, independent to its protease activity (Suga et al., 2006). *X. laevis* and *X. tropicalis* diverged from each other around 48 million years ago (Ma) before the divergence of the two diploid species that gave rise to the *X. laevis* subgenomes L and S, which occurred around 34 Ma (Session et al., 2016). Analysis of transposable elements (TE) or ‘jumping genes’ specific for each subgenome provided an evolutionary view of the two diploid ancestral species. These two species are still unknown. Specific TEs on each subgenome were active until 18 Ma suggesting that the allotetraploidization happened around 17 Ma. The *X. laevis* genome is composed of two homoeologous subgenomes called L and S for long and short, respectively (Session et al., 2016). The subgenome S is shorter due to more deletions than L. They have similar chromosome sizes to the *X. tropicalis* genome, suggesting they have ancestral chromosome organisation. In *X. laevis* eight out of the nine sets of chromosome pairs (S and L) have an orthologous chromosome in *X. tropicalis* and the ninth set of chromosome pairs is the result of a fusion of chromosomes 9 and 10 found in *X. tropicalis*. No inter-chromosomal rearrangements are observed in these two amphibian species (Session et al., 2016).

In this work, we show by a phylogenetic study that the ADAMTS family is conserved between Xenopus, human and mouse suggesting possible conservation of their functions. In addition identified *ADAMTS* genes in this study were not yet annotated and/or allocated on chromosomes in the current genome version. By analyses of the position of their homologues in *X. laevis* subgenomes and their orthologues in *X. tropicalis*, they were assigned to their corresponding chromosome contributing to improvement of Xenopus genomes assembly and annotation. We further show the spatio-temporal expression profiles of ADAMTS9 in *X. tropicalis* and *X. laevis*, showing them to be comparable to each other and to ADAMTS9 in mouse, reinforcing the possible conserved function between these species (Jungers et al., 2005).

## 2 Results

### 2.1 *ADAMTS* genes identification and phylogenetic study in *Xenopus laevis* and *Xenopus tropicalis*

**Fig. 2:**
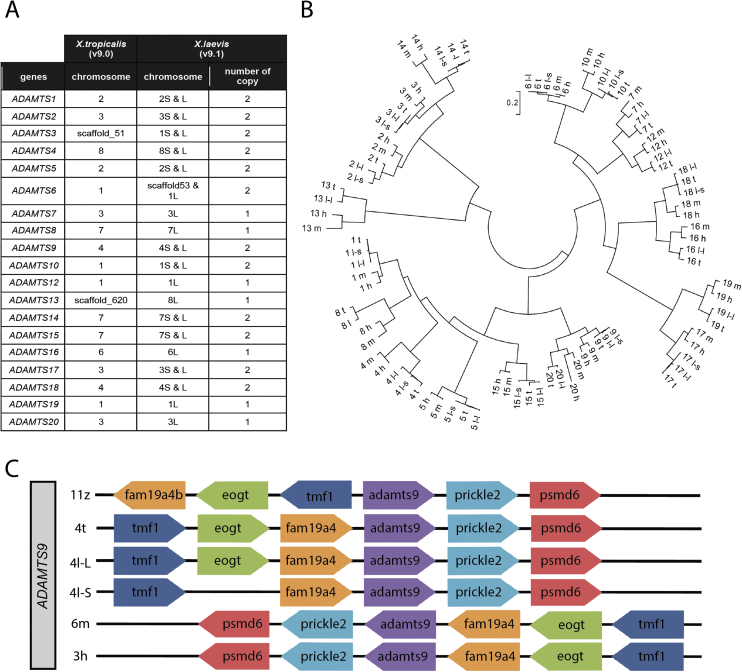
(A) Chromosomal location of ADAMTS genes in *Xenopus laevis* and *Xenopus tropicalis*. Chromosome number followed by an L for the longer and an S for the shorter indicate *X. laevis* gene location on the two subgenomes. Scaffold number is indicated for the genes without a chromosomal location. (B) Evolutionary relationships of the ADAMTS proteins. The evolutionary history was inferred by using the Maximum Likelihood method based on the JTT matrix-based model (Jones et al., 1992). The tree with the highest log likelihood (−21183.6512) is shown. Initial tree(s) for the heuristic search were obtained automatically by applying Neighbour-Join and BioNJ algorithms to a matrix of pairwise distances estimated using a JTT model, and then selecting the topology with superior log likelihood value. The tree is drawn to scale, with branch lengths measured in the number of substitutions per site. The analysis involved 88 amino acid sequences. All positions containing gaps and missing data were eliminated. There were a total of 355 positions in the final dataset. ADAMTS proteins are indicated by a number followed by a single letter code indicating the species: for example, ADAMTS1 t represents 1 from *Xenopus tropicalis*; 2 l-l and 2 l-s represent ADAMTS2 from *Xenopus laevis* subgenome L and subgenome S respectively; 3 h is ADAMTS3 from *Homo sapiens*; 4 m is ADAMTS4 from *Mus musculus*. (C) Synteny of *ADAMTS9* in zebrafish, *X. laevis* subgenomes L and S, *X. tropicalis*, *Mus musculus* and *Homo sapiens* genomes. Chromosome number is indicated on the left followed by a z for zebrafish, an l with L and S for the longer and shorter *X. laevis* subgenomes, respectively, a t for *X. tropicalis*, a m for *M.musculus* and a h for *H.sapiens*.

Protein sequences of the 19 human and mouse *ADAMTS*s were used as a reference to identify the *ADAMTS* genes in *X. laevis* and *X. tropicalis* using the UCSC Genome Browser. Genes with the highest percentage of similarity were selected. All 19 *ADAMTS* genes were present in both *X. tropicalis* and *X. laevis* genomes including seven singletons. *ADAMTS7*, *ADAMTS8*, *ADAMTS12*, *ADAMTS13*, *ADAMTS16*, *ADAMTS19* and *ADAMTS20* have only one copy in the *X. laevis* genome located on the L chromosome. The chromosomal position of *ADAMTS* genes is conserved between *X. tropicalis* and *X. laevis* confirming that no inter-chromosomal rearrangements happened in these species. *X. tropicalis ADAMTS3* and *ADAMTS13* and *X. laevis ADAMTS6S* are not allocated on chromosomes in the genome assembly. However, by analysis of their position on the set of chromosome pairs in *X. laevis* subgenomes and on the orthologous chromosome in *X. tropicalis* their positions are on chromosomes 1, 8 and 1S respectively (Fig. 2A).

*X. laevis* and *X. tropicalis* ADAMTSs were compared to ADAMTSs of *Mus musculus* and *Homo sapiens* by phylogenetic study using Maximum Likelihood method to determine their relations. Each *X. laevis* and *X. tropicalis* ADAMTS form a cluster on the same branch of the phylogenetic tree as the ADAMTS orthologues in *H. sapiens* and *M. musculus* with little divergences showed by the length of the branches suggesting possible conservation of their functions (Fig. 2B).

It has been shown that more gene deletions occurred on the *X.laevis* subgenome S than on the subgenome L, explaining the shorter size of the S subgenome compare to L and the *X. tropicalis* genome (Session et al., 2016). Synteny analysis was used to look at these events around the *ADAMTS9* gene. Locations and orientations of neighbour genes were compared between the three genomes. In order to carry out the synteny analysis, genes close to the gene of interest were selected. Due to the lack of annotation and/or assembly of scaffolds into chromosomes in the *X. laevis* (v9.1) and *X. tropicalis* (v9.0) genomes, neighbour genes were selected based on the criteria that they had to be annotated on the three genomes, even though they might not be the closest gene to the *ADAMTS* gene of interest. *X. laevis* subgenome L and *X. tropicalis* genomes have been shown to be more similar to each other than the *X. laevis* subgenome S. The synteny study of the three copies of *ADAMTS9* genes on these genomes shows that they have their chromosomal organisation conserved between *X. tropicalis* and *X. laevis* subgenome L but not with subgenome S due to loss of genes in the subgenome S (Fig. 2C). Comparison to the synteny maps of equivalent region in zebrafish, mouse and human show a closer conservation between Xenopus and mouse and human than to zebrafish.

### 2.2 *ADAMTS* family expression in *Xenopus tropicalis* and *Xenopus laevis*

**Fig. 3:**
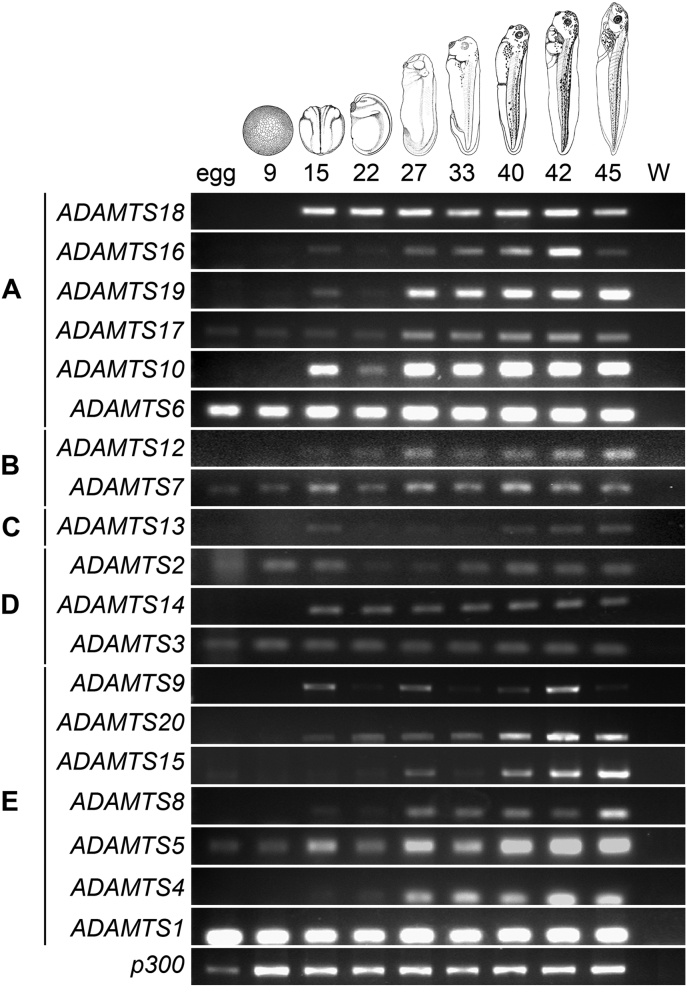
Expression of the *ADAMTS* genes family during *Xenopus tropicalis* development. Analysis by RT-PCR of mRNA from egg to stage 45. *Xenopus* stages are indicated at the top according to (Nieuwkoop and Faber, 1994) (NF) and gene names are indicated on the left. W: water (H_2_0), is a negative control and p300 is a loading control. *ADAMTS* genes are organised by clade as follow, (A) orphan, (B) COMP proteinases, (C) vWFCP, (D) Procollagen N-propeptidase and (E) Hyalectanases.

To look at the expression profiles of the *ADAMTS* family in Xenopus tropicalis during development the RNA of different whole embryos (n = 10) at key stages of development such as unfertilised egg, blastula (stage 9), neurula (stage 15), organogenesis at early tailbud (stage 22), tailbud (stage 27) and late tailbud (stage 33), and organogenesis at tadpole (stages 40, 42 and 45) was extracted (Fig. 3). cDNA synthesis was then carried out and the expression profiles established by RT-PCR. The same number of PCR cycles (35 cycles) was used for all *ADAMTSs* genes; *p300* was used as a loading control and H20 as a negative control. The signal intensity was different for the members of the *ADAMTS* family. *ADAMTS1*, *ADAMTS6* and *ADAMTS10* were the most strongly expressed whereas *ADAMTS2* and *ADAMTS13* were expressed at lower levels throughout development (Fig. 3). The maternal expression was measured at the unfertilised egg stage as the embryonic transcription only begins at the mid blastula transition (stage 8). Only *ADAMTS17*, *ADAMTS6*, *ADAMTS7*, *ADAMTS2*, *ADAMTS3*, *ADAMTS5* and *ADAMTS1* had maternal expression (Fig. 3). The expression of the *ADAMTSs* is thus highly dynamic across *X. tropicalis* developmental stages (Fig. 3).

The focus for the rest of this study is on the hyalectanases family composed of ADAMTS1, ADAMTS4, ADAMTS5, ADAMTS8, ADAMTS9, ADAMTS15 and ADAMTS20, during Xenopus development. Hyalectans, a subfamily of proteoglycans, have been shown to be essential for development (Brunet et al., 2012) and the different members of this family, aggrecan, versican, neurocan and brevican, can be cleaved by the hyalectanases family of ADAMTSs (Stanton et al., 2011).

**Fig. 4:**
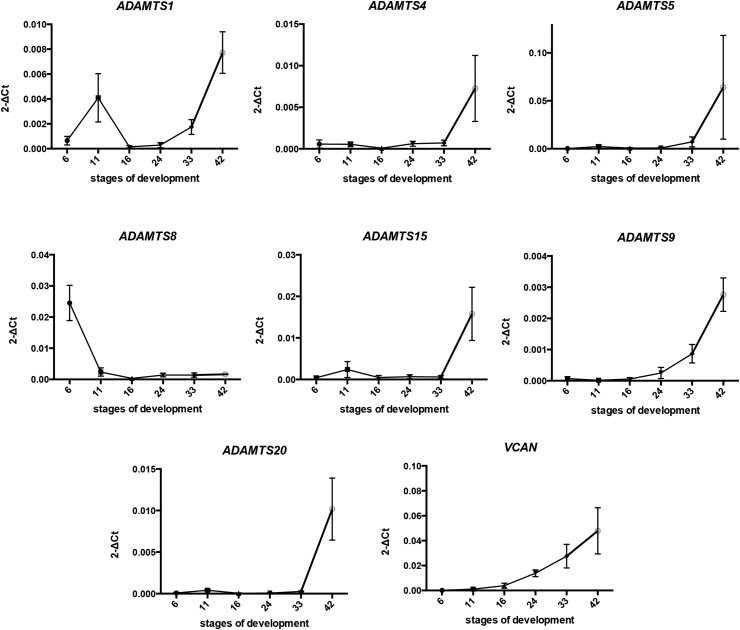
Expression profiles of the hyalectanases family and *VCAN* during *Xenopus laevis* development. Analysis by qRT-PCR of mRNA from stage 6 to stage 42 embryos normalized to odc as a loading control. The experiment was done three independent times. Each graph represents the expression profile of a single gene; the numbers on the vertical axis represents the normalized expression value and on the horizontal axis represents stages of development according to Nieuwkoop and Faber (1994) (mean with SEM), n = 3.

As RT-PCR is only a semi-quantitative method, quantitative RT-PCR (qRT-PCR) was used in order to look at the quantitative expression profiles of the hyalectanases during *Xenopus laevis* development. RNA from key developmental stages such as blastula (stage 6), gastrula (stage 11), neurula (stage 16), organogenesis at early tailbud (stage 24), late tailbud (stage 33) and tadpole (stage 42) was extracted. cDNAs synthesised from the RNA of these samples were used for qRT-PCR for the hyalectanases gene family and for versican (*VCAN*) one of their substrates (Fig. 4). Ct data from qRT-PCR were collected using 7500 Software v2.3 (Applied Biosystems) and analysed using Excel (Microsoft). ΔCt (gene Ct—odc Ct) were obtained from Ct values of each gene for normalization and then ΔCt values were converted to relative gene expression using the 2-ΔCt method. The 2-ΔCt method was used to determine the individual expression profiles using Graph Pad Prism 6 software (Fig. 4). The expression was low at early stages and high at late organogenesis stages (stage 42) for nearly all hyalectanase family members with the exception of *ADAMTS1* that showed a peak of higher expression at stage 11 (gastrula) and *ADAMTS8* that showed a peak of expression at stage 6 (blastula) suggesting a maternal expression (Fig. 4). The gene expression results for the hyalectanases gene family obtained by RT-PCR in *Xenopus tropicalis* (Fig. 3) showed similar higher expression at late stages of development compared to early stages as observed by qRT-PCR in *Xenopus laevis* (Fig. 4). Interestingly, *ADAMTS9* and its substrate, *VCAN*, showed the same expression profile with an expression increase at neurula (stage 16) to late organogenesis (stage 42) (Fig. 4).

### 2.3 *ADAMTS9* expression in *Xenopus tropicalis* and *Xenopus laevis*

**Fig. 5:**
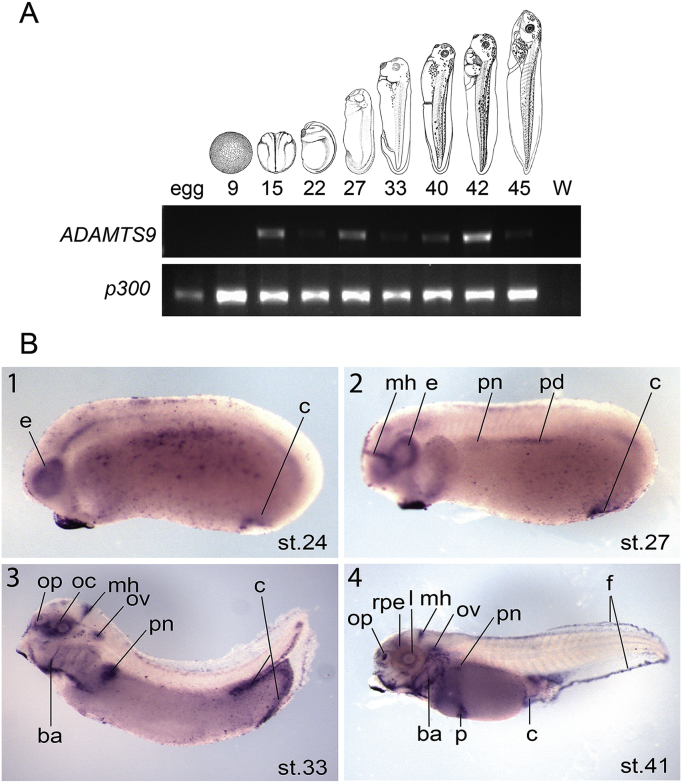
Expression of *ADAMTS9* during *Xenopus tropicalis* development. (A) Analysis by RT-PCR of mRNA from egg to stage 45. Xenopus stages are indicated at the top according to (Nieuwkoop and Faber, 1994) (NF) and gene names are indicated on the left. W: water (H20), is a negative control and p300 is a loading control. (B) Analysis by WISH of *ADAMTS9* expression. Developmental stages are according to (Nieuwkoop and Faber, 1994). Lateral views of embryos with anterior on the left and posterior on the right. (1) At stage 24 the expression is in the eye (e) and in the cloaca (c). (2) At stage 27 the expression is in the eye, in the midbrain hindbrain boundary (mh), in the pronephros (pn) and in the pronephric duct (pd) and in the cloaca (c). (3) At stage 33 the expression is in the olfactory placode (op), in the optic cup (oc), in the midbrain hindbrain boundary, in the otic vesicle (ov), in the pronephros, in the branchial arches (ba) and in the cloaca. (4) At stage 41 the expression is also in the lens (l), in the retinal pigment epithelium (rpe) in the pancreas (p) and in the fin (f).

**Fig. 6:**
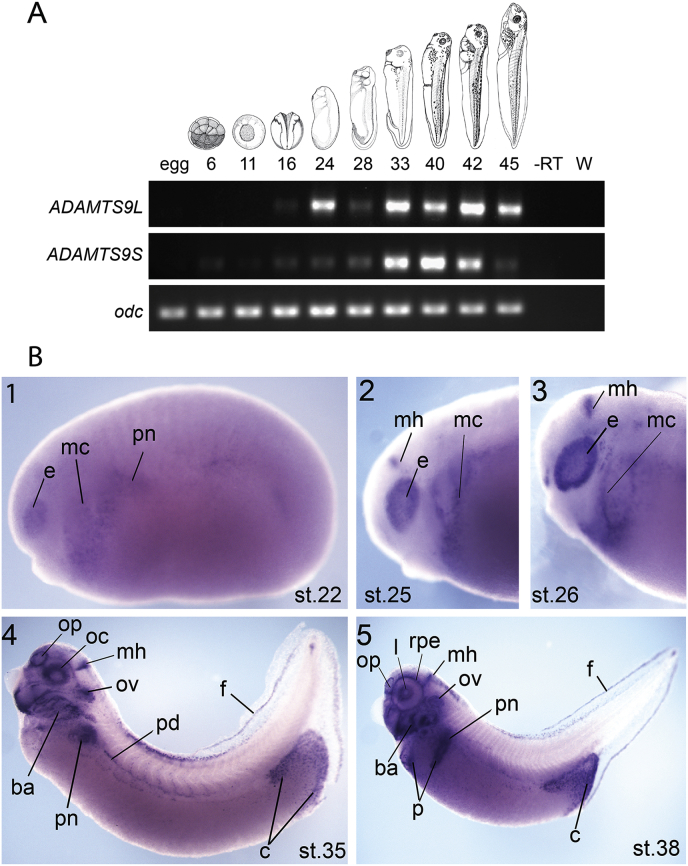
Expression of *ADAMTS9* during *Xenopus laevis* development. (A) Analysis by RT-PCR of mRNA from egg to stage 45. *Xenopus laevis* stages are indicated at the top according to (Nieuwkoop and Faber, 1994) (NF) and gene names are indicated on the left. odc is a loading control. -RT is a negative control of cDNA synthesis reaction made without adding the Reverse Transcriptase enzyme and W: water (H20), is a negative control of the PCR reaction without cDNA template. (B) Analysis by WISH of *ADAMTS9* expression. Developmental stages are according to Nieuwkoop and Faber, (1994). Lateral views of embryos with anterior on the left and posterior on the right. (1) At stage 22 the expression is in the eye (e), in migrating cells (mc) and in the pronephric anlage (pn). (2, 3) At stage 25 and 26 the expression is in the eye, in migrating cells (mc) and in the midbrain hindbrain boundary (mh). (4, 5) At stage 35 and 38 the expression is in the olfactory placode (op), in the optic cup (oc), in the midbrain hindbrain boundary, in the otic vesicle (ov), in the pronephros (pn), in the pronephric duct (pd), in the branchial arches (ba), in the fin (f) and cloaca (c). (5) At stage 38 the expression is also in the lens (l), in the retinal pigment epithelium (rpe), the fin and in the pancreas (p).

RT-PCR analysis in *X. tropicalis* and *X. laevis* show that *ADAMTS9* is not expressed in early stages before neurula and early tailbud stages (Fig. 5, Fig. 6). Indeed *ADAMTS9* starts to be expressed at stage 16 in *X. laevis* and stage 15 in *X.tropicalis*. *ADAMTS9S* in *X.laevis* starts to be expressed very weakly at stage 6 and goes up from stage 16. The strongest expression of *ADAMTS9* in *X. laevis* is found during organogenesis at late tailbud and tadpole stages (from stage 24 to stage 45) whereas in *X. tropicalis ADAMTS9* is more expressed at specific stages during organogenesis such as stage 27 and 42. *ADAMTS9S* expression in *X. laevis* is weaker than *ADAMTS9L* expression except at specific stages during organogenesis (stage 33, 40 and 42) (Fig. 5, Fig. 6A).

We next looked at spatial expression using wholemount in situ hybridisation (Fig. 5, Fig. 6B). In *X. tropicalis ADAMTS9* expression at early tailbud stages (stages 24) was mostly found in the developing eye and in the cloaca (Fig. 5B-1). At tailbud stages (stages 27), expression was observed in the eye, in the midbrain-hindbrain boundary, in the pronephros and in the pronephric duct and in the cloaca (Fig. 5B-2). At stage 33 *ADAMTS9* expression was seen in a number of developing organs and structures including the pronephros, the pronephric duct, the olfactory placode, the optic cup, the otic vesicle, the branchial arches and the cloaca (Fig. 5B-3). At tadpole stages (stages 41) the eye is more defined and the *ADAMTS9* expression was seen to be more specific to the lens and the retinal pigment epithelium. Expression was also observed in the pancreas and in the fin (Fig. 5B-4).

In *X. laevis ADAMTS9* expression at early tailbud stages (stages 22) was mostly seen in the developing eye, in migrating cells localised in the anterior of the embryo possibly corresponding to the heart field and in the pronephric anlage (Fig. 6B-1). At tailbud stages (stages 25 and 26) expression was observed in the eye, in the midbrain hindbrain boundary, in migrating cells localised on the anterior side of the embryo (Fig. 6B–2 and 3) and in the cloaca as observed in Fig. 5B-3 (data not shown). At stage 35 *ADAMTS9* expression was seen in developing organs and structures such as the pronephros, the pronephric duct, the olfactory placode, the optic cup, the otic vesicle, the branchial arches, the fin and the cloaca (Fig. 6B-4). At tadpole stages (stages 38) the eye structure was more defined and the *ADAMTS9* expression was specific to the lens and the retinal pigment epithelium, the expression was also seen in the pancreas, in the fin and in the cloaca (Fig. 6B-5).

**Fig. 7:**
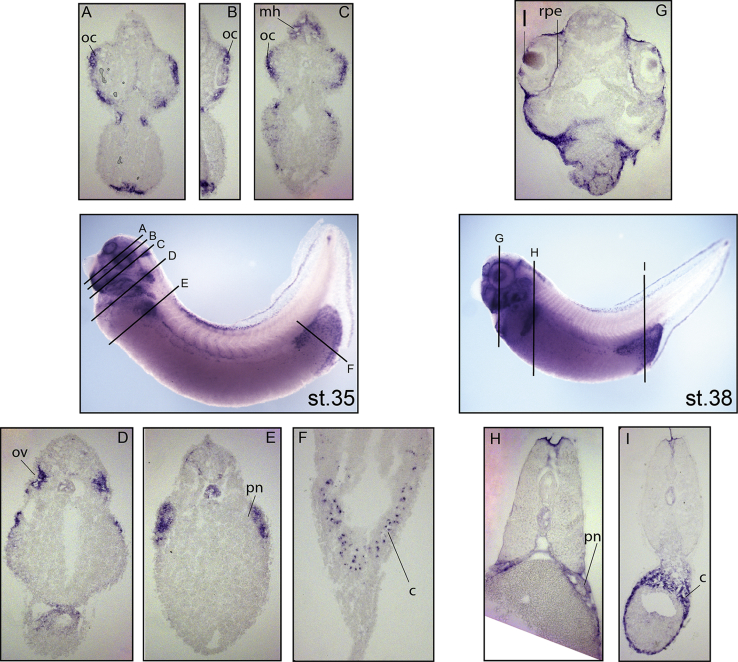
Sectioned *Xenopus laevis* embryos after WISH for *ADAMTS9*. Developmental stages are according to (Nieuwkoop and Faber, 1994). In each panel lines and numbers indicate the level of the sections. Embryos are shown with anterior on the right and sections with dorsal at the top. At stage 35, (A, B, C) the expression in in the head the expression is in the optic cup (oc), in the midbrain hindbrain boundary (mh) and (D) in the otic vesicle (ov); in the trunk (E, F) the expression is in the pronephros (pn) and in the cloaca (c). At stage 38, (G) in the head the expression is in the lens (l) and in the retinal pigment epithelium (rpe); in the trunk (H, I) the expression is in the pronephros (pn) and in the cloaca (c).

To look in more detail at the *ADAMTS9* expression profiles the head and the trunk of late *X. laevis* tailbud and tadpole embryos (stages 35 and 38) were sectioned (Fig. 7). In the head, expression was found in the optic cup, midbrain hindbrain boundary, in the otic vesicle at stage 35 (Fig. 7–A-D), in the lens and the retinal pigment epithelium at stage 38 (Fig. 7-G). In the trunk the expression was mostly found in the pronephric anlage at stage 35 and in the pronephros at stage 38 (Fig. 7–E and H) (Lienkamp et al., 2012).

## 3 Discussion

Xenopus diverged from mammals some 360 million years ago and has been shown to be a good animal model relative to human as they are both tetrapod, their genomes show conserved synteny and their organ development and function are highly similar (Wheeler and Brandli, 2009). Xenopus is a good model to study ECM remodeling during embryonic development because it can be seen externally unlike in mammals where embryonic development is in utero. This advantage allows a good visualisation of the phenotypes due to the disruption of a gene, by gain or loss of function experiments, and can help to define the timing of the apparition of these phenotypes.

19 *ADAMTS* genes are found in the *Xenopus laevis* subgenome L and *Xenopus tropicalis*, the same as in mammals, whereas only twelve are present in the subgenome S due to a loss of copy for seven *ADAMTS*s (*ADAMTS7*, *ADAMTS8*, *ADAMTS12*, *ADAMTS13*, *ADAMTS16*, *ADAMTS19* and *ADAMTS20*). Loss of these copies can be explained by neofunctionalization and/or regulation of the level of expression required during development in a stage and tissue dependant manner to adapt to environmental changes (gene dosage) (Otto, 2007; Suzuki et al., 2016). *X. tropicalis* and *X. laevis ADAMTS* chromosomal position is conserved, thus confirming the absence of interchromosomal rearrangements. It is known that 92% of the genes are conserved between *X. tropicalis* and the subgenome L of *X. laevis* when only 68% are conserved between *X. tropicalis* and the subgenome S of *X. laevis* (Session et al., 2016). The Xenopus *ADAMTS* gene family have the following proportions: 100% are present in *X. tropicalis* and *X. laevis* subgenome L but only 63% are present in *X. laevis* subgenome S. Some of the *ADAMTS* genes used in this study are not located on chromosomes or have not been annotated thus these data can help improve the genome assembly by allocating *ADAMTS* genes into the correct chromosome (Watanabe et al., 2016). The phylogenetic study shows that the ADAMTS family is conserved between Xenopus, human and mouse suggesting possible conservation of their functions.

The expression profile of the *ADAMTS* family in *Xenopus* embryonic development showed that all the members of the *ADAMTS* family are expressed in *Xenopus tropicalis*, visualised by RT-PCR, and have dynamic profiles showing variation in time and intensity of expression from egg to tadpole stages (stage 45). The hyalectanases family of ADAMTSs were the focus for the rest of the study as their substrate, the hyalectan/lectican family of chondroitin sulfate proteoglycans, are known to be widely expressed in the extracellular matrices during development (Brunet et al., 2012). To have a more precise view of the expression profiles of *ADAMTS1*, *ADAMTS4*, *ADAMTS5*, *ADAMTS8*, *ADAMTS9*, *ADAMTS15*, *ADAMTS20* and *VCAN* during *Xenopus laevis* development qRT-PCR was carried out from blastula (stage 6) to tadpole (stage 42) stages. High-resolution RNA-seq time course, showing transcripts per embryo, during *Xenopus tropicalis* embryonic development was published in open access in 2016 (Owens et al., 2016) allowing the comparison of published profiles of the hyalectanase family with the data obtained by qRT-PCR. The expression profiles were conserved for all of them (*ADAMTS4*, *ADAMTS5*, *ADAMTS15*, *ADAMTS9* and *ADAMTS20*) except for *ADAMTS8* that showed a high expression at stage 6 (maternal expression) in *Xenopus laevis* but not in Xenopus *tropicalis* and *ADAMTS1* that showed a peak of expression during gastrulation (stage 11). It has been shown that *ADAMTS1* plays a role in *Xenopus laevis* development at blastula to gastrula stage as a negative regulator of FGF by its C-terminal region, independent to its protease activity (Suga et al., 2006).

ADAMTS9, a member of the hyalectanases family, was the focus of the study as its expression was found very similar to the expression of its substrate versican by RT-PCR during Xenopus development suggesting a role of ADAMTS9 in the remodeling of this ECM component*. ADAMTS9* spatio-temporal expression profiles, assessed by RT-PCR and WISH, are similar in *Xenopus tropicalis* and *Xenopus laevis* from unfertilised egg to tadpole stages. *ADAMTS9* is highly expressed from early tailbud in the eye, the midbrain hindbrain boundary, and the migrating cells along the branchial arches (possibly neural crest cells). At tadpole stages *ADAMTS9* expression was seen in the retinal pigment epithelium, in the branchial arches, in the pronephros, in the pronephric duct and in the pancreas. *ADAMTS9* expression in the brain, the craniofacial structures, the kidney and the pancreas are similar for mouse and Xenopus (Jungers et al., 2005). Mice homozygous for a mutation in ADAMTS9 gene, leading to a knockout, do not survive past gastrulation (E7.5). However conditional and heterozygous mice for ADAMTS9 mutation survive and show soft tissue syndactyly (STS), when two or more digits are fused together and cardiac defects, respectively (Dubail et al., 2014; Kern et al., 2010).

It has been shown that *VCAN*, a substrate of ADAMTS9, was expressed in *Xenopus laevis* at early stages during gastrulation and neurulation, and at tailbud and tadpole stages the expression was found in the migrating neural crest, in the branchial arches, heart and pronephros (Casini et al., 2008). The co expression of *ADAMTS9* and its substrate *VCAN* in the pronephros, pronephric duct, migrating neural crest and their derivative structures, such as the branchial arches, suggest a role of ADAMTS9 in the extracellular matrix remodeling during the development of these structures by cleavage of versican (Stanton et al., 2011; Somerville et al., 2003). The cleavage of versican at the Glutamic acid (GLU) 441- Alanine (ALA) 442 position in the GAGβ domain generates a fragment containing the G1 domain and exposing the neo-epitode DPEAAE at the C-terminal position. This shorter versican fragment is called ‘versikine’. These smaller fragments of proteoglycans generated after proteolysis by the hyalectanses can have a biological function different from the intact form such as promoting apoptosis during interdigital web regression by decreasing the threshold of BMP required to induce apoptosis (McCulloch et al., 2009a). It has been shown by in vivo and in vitro study that versican is a nonpermissive matrix for NC migration leading them in the correct migratory direction by creating restrictive boundaries. Correct confinement is essential for collective NC migration during Xenopus development (Szabo et al., 2016). V0 and V1 are the most abundant versican isoforms found in neural crest cells (Dutt et al., 2006). Our expression analysis suggests a role of the hyalectanases family of ADAMTSs, such as ADAMTS9, in NC migration by potentially remodeling of the ECM by degradation of versican. In addition when we carried out chemical screens looking for compounds that affect pigment cell/melanophore development we identified a compound (NSC 84093) which affected melanophore migration by inhibiting metalloproteinase activity thus highlighting the importance of matrix remodeling (Tomlinson et al., 2009a, 2009b). MMP-2 and MMP-14 were identified as potential targets of this compound, however knock-down of these genes with morpholinos did not give rise to the same phenotype raising the hypothesis than other metalloproteinases might be involved in melanophore migration such as the ADAMTSs (Tomlinson et al. (2009a). Our results suggest that ADAMTS9 could be a good candidate. The same profile of expression of VCAN has been shown in mouse kidney development but the amount of cleaved versican, at the specific ADAMTSs cleavage site, was not affected by the knockout of ADAMTS1 and ADAMTS4 (Boerboom et al., 2011), suggesting the role of other members of the hyalectanases family, which could possibly include ADAMTS9.

ECM remodeling by ADAMTS9 modifying matrix dynamics can also have an effect on signaling pathways such as Shh and PDGFRb during umbilical cord vascular growth (Nandadasa et al., 2014). ADAMTS9 expression is found in the midbrain hindbrain boundary (MHB) from tailbud stages (Fig. 5, Fig. 6) whereas its substrate VCAN is not expressed in this area (Casini et al., 2008). FGF8 is known to be expressed in the MHB and to be essential for MHB development (Fletcher et al., 2006). ADAMTS1 has been shown to negatively regulate FGF independently to its catalytic activity (Suga et al., 2006). ADAMTS9 could play a role in the regulation of FGF signaling in the MHB development independently of its protease activity.

In order to test these hypothesis loss of function experiments using morpholinos or Crispr/Cas technology will have to be done in Xenopus embryos (Tomlinson et al., 2008; Guo et al., 2014).

In summary our work show that all the *ADAMTSs* members are expressed during both *Xenopus laevis* and *Xenopus tropicalis* development with different dynamic of expression during embryogenesis. In particular ADAMTS9 presents an interesting expression pattern, similar to its potential substrate versican suggesting a role either in matrix remodeling by removal of a versican scaffold and/or generation of cleavage products such as versikine.

### 3.1 Experimental procedures

All experiments were performed in compliance with the relevant laws and institutional guidelines at the University of East Anglia. The research has been approved by the ethics committee of the University of East Anglia.

### 3.2 Data source of ADAMTS gene sequences

The ADAMTS genes reported by (Brocker et al., 2009) were retrieved from Genbank (http://www.ncbi.nlm.nih.gov). With these genes as queries, the Basic Local Alignment Search Tool (BLAST) program was used to search in National Center for Biotechnology Information (NCBI) and in The UniProt Knowledgebase (UniProtKB) against *M.musculus* and *H.sapiens* genome assemblies. Orthologs of known human *ADAMTS* genes in Xenopus were identified by using BLAT sequence-based searches of the Xenopus genomes hosted at The Francis Crick Institute (UCSC Genome Bioinformatics Site). The version xenTro9.1 assembly was used for *Xenopus tropicalis* and xenLae2 assembly was used for *Xenopus laevis*. Nucleotide sequences were translated to a protein sequence using ExPASy - Translate tool.

### 3.3 Alignment and phylogenetic analysis

Multiple sequence alignments and phylogenetic trees were constructed using the amino acid residues of the full length genes and were generated using MEGA (Molecular Evolutionary Genetics Analysis) version 6 software (Tamura et al., 2013).

### 3.4 Reverse transcriptase-polymerase chain reaction (RT-PCR) and quantitative RT-PCR (qRT-PCR)

*Xenopus laevis* embryos were obtained as previously described (Harrison et al., 2004). Total RNA from embryos at the indicated stages was extracted using TRIzol^®^ (15596-026, Life technologies, UK) and DNAse treated. Reverse transcription was performed using SuperScript^®^ II reverse transcriptase (18064-014, Invitrogen, CA, USA), with 1 μg total RNA and random primers (C1181, Promega, Southampton, UK).

The amplification of templates was performed using a thermocycler. Total PCR reaction was 25 μl containing 10–50 ng of template cDNA, 0.2 μM of each forward and reverse primer, 1× of BioMix™ (2× reaction mix containing ultra-stableTaq DNA polymerase, Bioline). An initial denaturation step of 95 °C for 3 min was followed by a denaturation of 1 min at 95 °C. The annealing step was carried out at an annealing temperature calculated by subtracting 5 °C from the primer melting temperature, for 1 min. This was followed by 1 min of extension step at 72 °C (according to the expected size of the PCR product, 30s/500bp). 25–35 cycles (depending on the level of expression of the gene of interest) of denaturation, annealing and extension were carried out. Amplified products were fractionated in 1% (w/v) agarose Tris/Borate/EDTA (TBE 1×: 45 nM Tris-Borate, 1 mM EDTA, pH8.0) gel electrophoresis with 0.0001% (v/v) of 10 mg/ml ethidium bromide and visualised under UV light using a UV transilluminator (BIO-RAD).

Sequences and terms of use of each primer set for RT-PCR in *Xenopus tropicalis* are indicated below. The *ADAMTS9* primers were used to make the in situ probe.

**Table:** 

*Xenopus tropicalis* gene name	Tm (°C)	Sense primer	Anti-sense primer	Reference
*ADAMTS1*	59.2	CTTTTCTTGCCCCGGACTTC	CTCGGTAGAAGAAGGCTCCC	PRIMER3/ SIGMA
*ADAMTS4*	59.2	GACCCCAGTTTTCTCTCCGA	TGCAGAACCCCAACAAGAGA	PRIMER3/ SIGMA
*ADAMTS5*	59.1	TACGCGGATGGGAAGAAGTT	TAATGGGCATGCTTGACTGC	PRIMER3/ SIGMA
*ADAMTS8*	58.9	GACCTTGCGATTTACTGCACT	CACCCTGAACACCTTTGCAT	PRIMER3/ SIGMA
*ADAMTS15*	59.2	CTATGCACTTCCCTGGCTCT	TATTCCTCCCCTTGGTAGCC	PRIMER3/ SIGMA
*ADAMTS9*	62	TTAGCAGTGGTCCATGATGAA	TTCCCGGCTCACATTCG	PRIMER3/ SIGMA
*ADAMTS20*	59	GGGCTTGTCTGTCATCTCT	AGGTGTGGTTTGTTGTGCTC	PRIMER3/ SIGMA
*ADAMTS2*	58.9	AGTTCCGGACAGTGAAGTGT	TGAGACCAAGGCCCAATTCT	PRIMER3/ SIGMA
*ADAMTS3*	59.1	AAAGCTGTGGAAGTTCTGGC	CAGACCATGCCCCTGTTTTC	PRIMER3/ SIGMA
*ADAMTS14*	59.1	AGCCAAAACCAATACGCAGA	TGGTGTCCGATTACAGGGTC	PRIMER3/ SIGMA
*ADAMTS13*	59	CAATGGGGTGGTACTGGAGT	GGGTTCCAGGTCCATGTACA	PRIMER3/ SIGMA
*ADAMTS7*	59	GGAGATGGTGGACAAGGGAA	GTGCCTCTGACTGGGTACTT	PRIMER3/ SIGMA
*ADAMTS12*	58.8	ATGAGCCGTGTGATTCCTCT	TGGTAAGAAGATGCGCCTCT	PRIMER3/ SIGMA
*ADAMTS6*	58.9	CCTTGTCATGGCTTCATCGG	ACTCCTCCTCCTCCTCAAGT	PRIMER3/ SIGMA
*ADAMTS10*	59	GAGGTCTGGACTGGAAGCAT	CTCCAAGTGTTGACGTTGCA	PRIMER3/ SIGMA
*ADAMTS16*	58.3	ACCAAGGAAGATTCAAACGATCA	TGTCAACACGTAGGTAGTAATGT	PRIMER3/ SIGMA
*ADAMTS18*	58.6	GAGATCAGCGCGAAGTTCAA	GGAAACCTGACATGCCATCA	PRIMER3/ SIGMA
*ADAMTS17*	58.9	ACAGAGGAGAGGGACCAAAC	TCCCTGGTTTTCTCCTCCAC	PRIMER3/ SIGMA
*ADAMTS19*	59.2	CAACCAACGCATCATCTCCC	GAAGATCCTCTCCACTCCCG	PRIMER3/ SIGMA

*Xenopus laevis* gene name	Tm (°C)	Sense primer	Anti-sense primer	Reference
*ADAMTS9S*	60	CACCCAAGAGCCAAGTTCCTAGACA	AGTGTGCTCTGTCCTGTCAT	PRIMER3/ SIGMA
*ADAMTS9L*	60	CACCGTGAATGCTGAATCCCGACC	TGTCATTCACTTGGCCACTG	PRIMER3/ SIGMA
*ADAMTS9* *(probe)*	58	TTAGCAGTGGTCCACGATGAA	AGGAACACATACTCCATATCTG	PRIMER3/ SIGMA

PCR products of ADAMTS9 in *Xenopus laevis* and Xenopus tropicalis were cloned into pGEMTeasy vector, sequenced and used as a template to generate sense and antisense in situ probes.

Quantitative RT-PCR was carried out as previously described (Hatch et al., 2016).

Sequences and terms of use of each primer set for RT-qPCR in *Xenopus laevis* are indicated below.

**Table:** 

*Xenopus laevis* gene name	Tm (°C)	Sense primer	Anti-sense primer	Reference
*ADAMTS1*	57	CAGGAGGCACGAGGAAGAA	ATGAGGGTGAGGAGATAATGTTTC	Primerdesign
*ADAMTS4*	56.6	CTATCGCCGCTATCAACTACAA	TGAGTCCTCCACCTTCCAAG	Primerdesign
*ADAMTS5*	57	GGGCAAGGTGGGCTACAT	CTGAAGTGGGGAGACAACAAC	Primerdesign
*ADAMTS8*	56.4	TTTAGTTCCTGATGATGCTTTTCTT	GCTGCCAGTGGTTCCATAC	Primerdesign
*ADAMTS15*	56.7	AATCCAATCAACATTGTCGTTGTG	TCAGTGTCATGGCGGCATT	Primerdesign
*ADAMTS9*	56.4	GCCCGACTGGAATACAATGAT	ATGTTTTGCGTTTTACTGAAGAGA	Primerdesign
*ADAMTS20*	56.8	TTAGAACCACTGATGAAACCTGAT	AATGTTTGACTTTGTGCTTGTAGA	Primerdesign
*VCAN*	56.8	TCATTATCTGGAAGAGTGAATCTGC	CATCTCTGCTCTGGACAATCTTG	Primerdesign

### 3.5 Wholemount in situ hybridizations

Wholemount in situ hybridisation was carried out as previously described (Harrison et al., 2004). Sense and antisense probes were synthesised for *ADAMTS9* in *Xenopus tropicalis* and *Xenopus laevis*. The ADAMTS9 probe in Xenopus laevis was designed to detect both copies of the gene in order to obtain a stronger signal.

### 3.6 Cryosectioning

Embryos were sectioned as previously described (Hatch et al., 2016).

## Funding

This work was supported by a BBSRC grant (BB/I022252) to GW and by the People Programme (Marie Curie Actions) of the European Union's Seventh Framework Programme FP7 under REA grant agreement number 607142 (DevCom) to ID.

## Conflict of interest

None.
